# Complete blood count-based inflammatory score (CBCS) is a novel prognostic marker for gastric cancer patients after curative resection

**DOI:** 10.1186/s12885-019-6466-7

**Published:** 2020-01-06

**Authors:** Jian-Xian Lin, Jun-Peng Lin, Jian-Wei Xie, Jia-bin Wang, Jun Lu, Qi-Yue Chen, Long-long Cao, Mi Lin, Ruhong Tu, Chao-Hui Zheng, Chang-Ming Huang, Ping Li

**Affiliations:** 1 Department of Gastric Surgery, Fujian Medical University Union Hospital, Fuzhou, Fujian Province China; 2 Department of General Surgery, Fujian Medical University Union Hospital, Fuzhou, Fujian Province China; 3 Key Laboratory of Ministry of Education of Gastrointestinal Cancer, Fujian Medical University, Fuzhou, Fujian Province China; 4 Fujian Key Laboratory of Tumor Microbiology, Fujian Medical University, Fuzhou, Fujian China

**Keywords:** Gastric cancer, Complete blood count, Biomarker, Prognosis

## Abstract

**Background:**

We sought to investigate the prognostic value of complete blood count (CBC)-based biomarkers for patients with resectable gastric cancer (GC).

**Methods:**

Patients with GC who underwent primary surgical resection between December 2008 and December 2013 were included. The estimated area under the curve (AUC) and multivariate Cox regression models were used to identify the best CBC-based biomarker. Time-dependent receiver operating characteristic (t-ROC) curve analysis was used to predict overall survival and compare the prognostic impact.

**Results:**

In the 1810 patients analyzed, the median follow-up period was 51.0 months (range 1–101 months). Based on multivariate analysis, the lymphocyte-monocyte ratio (LMR) and hemoglobin (Hb) level were independent prognostic factors (both *P* < 0.05). Based on the LMR and Hb level, we established the CBC-based inflammatory score (CBCS). A higher CBCS was associated with older age, female sex, higher American Society of Anesthesiologists (ASA) score, proximal tumor location, larger tumor size, later stage and vascular involvement (all *P* < 0.05). Univariate analyses showed that a higher CBCS was also associated with worse overall survival (OS), which was consistent in each stage (all P < 0.05). Multivariate analysis revealed that the CBCS was a significant independent biomarker (P < 0.05). The AUC for the CBCS (0.627) was significantly higher than the AUCs for the LMR (0.573) and Hb level (0.605) (both P < 0.05). Furthermore, the t-ROC curve of the CBCS was superior to that of the prognostic nutritional index (PNI), systemic immune-inflammation index (SII), modified Glasgow prognostic score (mGPS) and C-reactive protein/albumin ratio (CRP/Alb) throughout the observation period.

**Conclusion:**

The preoperative LMR and Hb level were optimal CBC-based biomarkers for predicting OS in GC patients after curative resection. Based on the LMR and Hb, we developed a novel and easily obtainable prognostic score called the CBCS, which may improve the prediction of clinical outcomes.

## Background

Gastric cancer (GC), one of the most common malignancies, is the third most common cause of cancer-related death worldwide [1]. Despite improvements in surgical techniques and therapeutic modalities, survival of GC remains poor [2]. The role of inflammation in the development of tumors was first described in the nineteenth century [3]. Currently, there is accumulating evidence that the host inflammatory response plays an important role in the development and progression of cancer [4]. Complete blood count (CBC)-based biomarkers are a series of inflammatory indicators based on blood cells [5]. Pretreatment CBC-based biomarkers, including blood neutrophil, lymphocyte, monocyte, and platelet counts; hemoglobin (Hb) levels; and their combinations, such as the neutrophil-lymphocyte ratio (NLR), lymphocyte-monocyte ratio (LMR) and platelet-lymphocyte ratio (PLR), have been reported to reflect systemic and local inflammation associated with cancer progression and prognosis [6–10].

The American Joint Committee on Cancer (AJCC) staging system is the most widely used system to assess prognosis [11], but survival can vary in patients with GC even when they have the same TNM stage. Therefore, it is necessary to improve the individual prognostic prediction for GC by combining the AJCC staging system with other prognostic indicators. Previous studies have shown that preoperative CBC-based biomarkers can be used as part of a prognostic model to predict the prognosis of tumors more accurately [12, 13]. However, there is likely some degree of overlap between these CBC-based biomarkers, and some may be redundant. Therefore, for future studies and potential clinical use, a more simple and effective prognostic inflammatory biomarker is required. The aim of this study was to further evaluate the prognostic value of these CBC-based biomarkers and to establish a simple inflammatory scoring system based on CBC biomarkers to efficiently predict long-term outcomes for GC patients after surgery.

## Methods

### Study population

Patients who consecutively underwent radical gastrectomy at Fujian Medical University Union Hospital from December 2008 to December 2013 were enrolled in this study. Our exclusion criteria were as follows: (1) no routine blood examination before surgery, (2) metastatic disease, (3) neoadjuvant chemotherapy, (4) malignant disease in other organs, and (5) incomplete/inaccurate follow-up data or postoperative pathological staging. Finally, 1810 patients were included in the study (Additional file 2: Figure S1). All surgical procedures, including D2 lymphadenectomy, were performed according to the guidelines of the Japanese Gastric Cancer Association [14]. Staging was performed according to the corresponding seventh edition of the AJCC Staging Manual [11]. Adjuvant chemotherapy using 5-fluorouracil (5-FU)-based regimens (mostly oxaliplatin with either Xeloda or S-1) was recommended for the majority of patients with advanced GC [15, 16].

### Definition of inflammation-based biomarkers

Patients routinely underwent blood testing during the 7 days before surgery [17]. The blood samples were usually sent directly to the laboratory for analysis within 1 h after blood extraction. These included CBC, Hb level, albumin (Alb) level, and C-reactive protein (CRP) level (where available). Candidate CBC-based biomarkers considered in our study included the Hb level, individual cell counts (absolute neutrophil, lymphocyte, monocyte, and platelet), cell count ratios (NLR, LMR, and PLR) and the systemic immune-inflammation index (SII). The SII is calculated as the platelet count × the NLR [9]; the PNI consists of the lymphocyte count and the albumin level [18]; and the modified Glasgow prognostic score (mGPS) consists of the CRP and albumin levels [19]. X-tile software (Yale University, New Haven, CT, USA) can be used to perform a time-dependent cutoff value analysis based on survival information [20], and it has been widely used in many previous studies [12, 21, 22]. X-tile software divided the population into different strata based on every possible cutoff point. All possible divisions based on the cutoff points were assessed. The optimal cut-off value for survival was calculated by selecting the minimum *P* value with the maximum χ^2^ value [20]. Therefore, the optimal cutoff values for the PLR, LMR, Hb level and SII were 161.3, 3.4, 125 g/l and 570, respectively, according to the X-tile software.

### Follow-up investigation

A postoperative follow-up assessment was performed every 3 months for 2 years and then every 6 months from years 2 to 5. The final follow-up evaluation was conducted in December 2017. Most routine follow-up appointments included a physical examination, laboratory testing (including measurements of the levels of cancer antigen [CA] 19–9, CA72–4, and carcinoembryonic antigen [CEA]), chest radiography, and abdominopelvic ultrasonography or computed tomography, along with an annual endoscopic examination. Overall survival (OS) was defined as the time from surgery to death from any cause or to the time of censoring on the date of the last follow-up.

### Statistical analysis

Descriptive statistics were used to summarize the cohort characteristics and the distributions of CBC-based predictors. To avoid issues with multicollinearity in ensuing analytic steps, we compared similar predictors and only retained the predictors with the superior estimated AUC for further evaluation [5]. Continuous variables were analyzed by Student’s t tests, and categorical variables were compared using the chi-square test or Fisher’s exact test. Survival curves were generated by the Kaplan-Meier method and analyzed using the log-rank test. Univariate and multivariate analyses were calculated by the Cox proportional hazards regression model [23]. Then, internal validation was performed by simple bootstrapping, applying resampling with replacement 10,000 times in the total cohort [24]. The “timeROC” package in R was used to generate time-dependent receiver operating characteristic (t-ROC) curves of the inflammatory scores. The t-ROC curve analysis is an extension of the ROC curve analysis, and it assesses the discriminatory power of continuous variables for time-dependent disease outcomes [25]. In addition, to compare the ROC curves, AUCs can be calculated [26]. Sequential AUCs were compared between two scores using independent and identically distributed representations of AUC estimators. Statistical significance was set at *P* < 0.05. Statistical analyses were performed using SPSS for Windows version 18.0 (SPSS Inc., Chicago, IL, USA) and R version 3.1.2 (R Foundation for Statistical Computing, Vienna, Austria).

## Results

### Clinicopathological characteristics

Of the 1810 GC patients included in the study, 1374 (75.9%) were male, and 436 (24.1%) were female, and their median age was 61 years (interquartile range (IQR): 55–69 years). The distribution of TNM stages was as follows: 515 (28.5%) patients had stage I disease, 433 (23.9%) had stage II disease, and 862 (47.6%) had stage III disease (Additional file 1: Table S1). The AUCs for 5-year OS were used to identify the best predictors among those that were similar to one another, as shown in Additional file 1: Table S2. The AUC for the PLR was superior to those for the absolute platelet counts, neutrophil counts, lymphocyte counts, NLR and SII, whereas the AUC for the LMR was superior to those for the absolute monocyte count and absolute lymphocyte count. There were no predictors similar to the Hb level. Thus, only the PLR, LMR, and Hb level were retained for further analyses.

### Survival analysis

The median follow-up period was 51.0 months (range 1–101 months). The 5-year OS rates for the entire cohort were 67.2%. The cutoff values for the PLR, LMR and Hb were 161.3, 3.4 and 125 g/l, respectively, as determined by the X-tile software. In the Kaplan-Meier analyses, a higher PLR (≥161.3), a lower LMR (< 3.4) and a lower Hb level (< 125 g/l) were found to be associated with worse OS (all *P* < 0.05, Additional file 2: Figure S2A-C). Univariate analysis showed that the CBC-based biomarkers associated with OS included the PLR, LMR and Hb level (all P < 0.05, Table 1). In addition, other variables, including age, body mass index (BMI), the American Society of Anesthesiologists (ASA) score, tumor location, tumor diameter, vascular invasion and perineural invasion, had significant effects on OS (all P < 0.05, Table 1). In multivariate analyses, the LMR (*P* = 0.003) and Hb level (P = 0.003) were independent CBC-based factors affecting the prognosis (Table 1).

**Table 1:** Univariate and multivariate analyses of clinicopathological variables in relation to overall survival in patients undergoing potentially curative resection for gastric cancer

Clinicopathological features	Univariate analysis		Multivariate analysis	
	HR (95% CI)	P	HR (95% CI)	*P*
Age	1.03 (1.02–1.04)	< 0.001	1.02 (1.01–1.03)	< 0.001
Sex		0.979		
Male	Reference			
Female	1.00 (0.83–1.21)			
BMI	0.94 (0.92–0.97)	< 0.001		0.058
ASA score		0.013		0.740
1	Reference			
2	1.29 (1.09–1.53)			
3	1.18 (0.75–1.86)			
Tumor location		< 0.001		< 0.001
Upper	Reference		Reference	
Middle	1.05 (0.83–1.33)		0.92 (0.72–1.17)	
Lower	0.59 (0.48–0.73)		0.79 (0.64–0.97)	
Mixed	1.56 (1.22–1.98)		1.32 (1.03–1.70)	
Tumor size (cm)	1.02 (1.01–1.02)	< 0.001	1.01 (1.00–1.01)	0.003
Histologic type		0.069		
Differentiated	Reference			
Undifferentiated	1.22 (0.99–1.51)			
Vascular invasion		< 0.001		0.655
Negative	Reference			
Positive	1.69 (1.41–2.02)			
Perineural invasion		< 0.001		0.885
Negative	Reference			
Positive	1.63 (1.33–1.99)			
pTNM stage		< 0.001		< 0.001
I	Reference		Reference	
II	2.65 (1.82–3.68)		2.00 (1.38–2.90)	
III	9.74 (7.05–13.46)		6.80 (4.91–9.41)	
Adjuvant chemotherapy		0.260		
No	Reference			
Yes	1.10 (0.93–1.31)			
Hb		< 0.001		0.003
< 125	Reference		Reference	
≥ 125	0.51 (0.43–0.60)		0.77 (0.65–0.91)	
LMR		< 0.001		0.003
< 3.4	Reference		Reference	
≥ 3.4	0.59 (0.50–0.70)		0.78 (0.65–0.91)	
PLR		< 0.001		0.434
< 161.3	Reference			
≥ 161.3	1.70 (1.44–2.00)			

### Establishment of the CBC-based inflammatory score (CBCS)

Based on the survival analysis above, we combined the LMR and Hb level and generated four subgroups. We found significant differences among the four subgroups (Additional file 2: Figure S2D). In subgroups with either LMR ≥ 3.4 or Hb ≥ 125 g/l, the OS was similar (*P* > 0.05, Additional file 2: Figure S2D). Thus, we combined those two subgroups to establish the CBCS as follows: patients with both an elevated LMR and an elevated Hb level (≥3.4 and ≥ 125 g/l, respectively) were assigned a score of 0; patients with either a reduced LMR or a reduced Hb level were assigned a score of 1, and patients with both a reduced LMR and a reduced serum Hb level (< 3.4 and < 125 g/l, respectively) were assigned a score of 2 (Additional file 1: Table S3).

Next, we analyzed the correlations between the CBCS and clinicopathological characteristics (Table 2). A higher CBCS was associated with older age, female sex and a higher ASA score (all *P* < 0.05, Table 2). Regarding tumor factors, proximal tumor location, larger tumor size, higher pTNM stage, and vascular invasion were significantly associated with a higher CBCS (all P < 0.05, Table 2).

**Table 2:** Relationship between the CBCS and clinicopathological characteristics in patients undergoing potentially curative resection for gastric cancer

Clinicopathological features	CBCS			*P* value
	0	1	2	
Case	824	673	313	
Age, median (IQR)	59	63	66	< 0.001
Sex				< 0.001
Male	668 (81.1%)	474 (70.4%)	232 (74.1%)	
Female	156 (18.9%)	199 (29.6%)	81 (25.9%)	
BMI, median, (IQR)	22.3	21.9	21.8	0.468
ASA score				< 0.001
1	578 (70.1%)	399 (59.3%)	149 (47.6%)	
2	238 (28.9%)	244 (36.3%)	144 (46.0%)	
3	8 (1.0%)	30 (4.5%)	20 (6.4%)	
Tumor location				0.001
Upper	195 (23.7%)	176 (26.2%)	70 (22.4%)	
Middle	119 (14.4%)	124 (18.4%)	77 (24.6%)	
Lower	405 (49.2%)	290 (43.1%)	120 (38.3%)	
Mixed	105 (12.7%)	83 (12.3%)	46 (14.7%)	
Tumor size (cm), median (IQR)	4.0	4.5	5.0	< 0.001
Histologic type				0.725
Differentiated	159 (19.3%)	141 (21.0%)	62 (19.8%)	
Undifferentiated	665 (80.7%)	532 (79.0%)	251 (80.2%)	
Vascular invasion				< 0.001
Negative	675 (81.9%)	513 (76.2%)	218 (69.6%)	
Positive	149 (18.1%)	160 (23.8%)	95 (30.4%)	
Perineural invasion				0.219
Negative	707 (85.8%)	575 (85.4%)	256 (81.8%)	
Positive	117 (14.2%)	98 (14.6%)	57 (18.2%)	
pTNM stage				< 0.001
I	323 (39.2%)	148 (22.0%)	44 (14.1%)	
II	179 (21.7%)	174 (25.9%)	80 (25.6%)	
III	322 (39.1%)	351 (52.2%)	189 (60.4%)	

### Correlations of the CBCS with survival rates

**Fig. 1:**
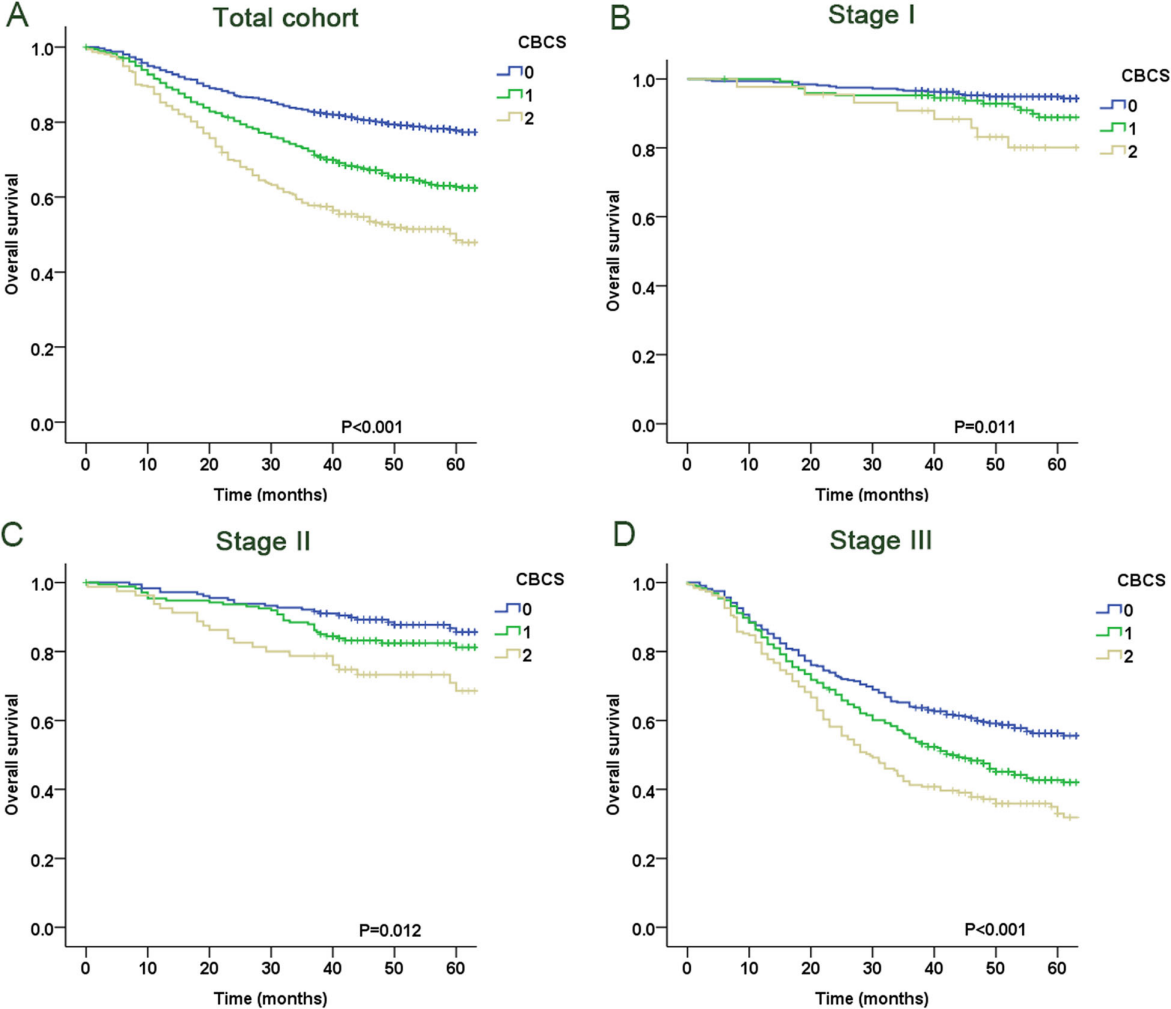
Kaplan-Meier analysis of overall survival according to SIS in the (**a**) total cohort and (**b**) stage I, (**c**) stage II, and (**d**) stage III subgroups

Kaplan-Meier curves for the 5-year OS were divided into 3 groups according to the CBCS (CBCS = 0: 77.8%, CBCS = 1: 62.7%, and CBCS = 2: 48.5%; log-rank test: P < 0.05, Fig. 1a). After adjusting for pTNM stage, the CBCS was strongly associated with the OS of patients in each stage of disease, including in the stage I, II and III subgroups (all P < 0.05, Fig. 1b-d). Multivariate analyses revealed that CBCS (*P* < 0.001), age (P < 0.001), tumor location (P < 0.001), tumor size (*P* = 0.003) and pTNM stage (P < 0.001) were associated with OS (Table 3). A prediction model was established by combining pTNM stage and the CBCS, and the AIC of the model was lower than that of pTNM stage (8066.9 vs. 8101.3), but the AUC value of the model was significantly better than that of pTNM stage (0.775 vs. 0.746, P < 0.001).

**Table 3:** Multivariate analysis of the CBCS clinicopathological variables in relation to overall survival in patients undergoing potentially curative resection for gastric cancer

Clinicopathological features	Multivariate analysis*		Internal validation*	
	HR (95% CI)	*P*	HR (95% CI)	*P*
Age	1.02 (1.02–1.03)	< 0.001	1.02 (1.02–1.03)	< 0.001
Tumor Location		< 0.001		< 0.001
Upper	Reference		Reference	
Middle	0.92 (0.72–1.17)		0.91 (0.72–1.15)	
Lower	0.79 (0.64–0.97)		0.78 (0.64–0.96)	
Mixed	1.32 (1.03–1.70)		1.30 (1.02–1.66)	
Tumor size (cm)	1.01 (1.00–1.01)	0.003	1.01 (1.00–1.01)	< 0.001
pTNM stage		< 0.001		< 0.001
I	Reference		Reference	
II	2.00 (1.38–2.91)		2.02 (1.42–2.88)	
III	6.81 (4.92–9.42)		6.82 (4.97–9.36)	
CBCS		< 0.001		< 0.001
0	Reference		Reference	
1	1.29 (1.06–1.57)		1.27 (1.03–1.54)	
2	1.68 (1.35–2.09)		1.60 (1.22–1.94)	

*Adjusted for the following variables: age, BMI, ASA score, tumor location, vascular invasion, perineural invasion, pTNM stage, CBCS, and PLR

Internal validation confirmed that the CBCS is an independent prognostic factor for GC (CBCS = 1: HR = 1.270; CACS = 2: HR = 1.604, P < 0.001), and other independent prognostic factors included age, tumor location, tumor size and pathological stage (all P < 0.001, Table 3).

### Comparison of the CBCS with its components (LMR and Hb) in terms of prognostic accuracy for the prediction of 5-year OS

The CBCS was based on the LMR and the Hb level (Additional file 1: Table S3). We thus explored the prognostic accuracies of the CBCS and each of its components—the LMR and Hb level—by generating AUCs for the prediction of 5-year OS. The AUCs for the CBCS, LMR and Hb were 0.627 (95% confidence interval [CI] 0.604–0.650), 0.573 (95% CI 0.549–0.596) and 0.605 (95% CI 0.582–0.628), respectively. The AUC for the CBCS was significantly higher than that for each component of the CBCS (both *P* < 0.05).

### Comparison of the CBCS with other inflammatory scoring systems (PNI, SII, mGPS and CRP/Alb) in terms of prognostic accuracy

**Fig. 2:**
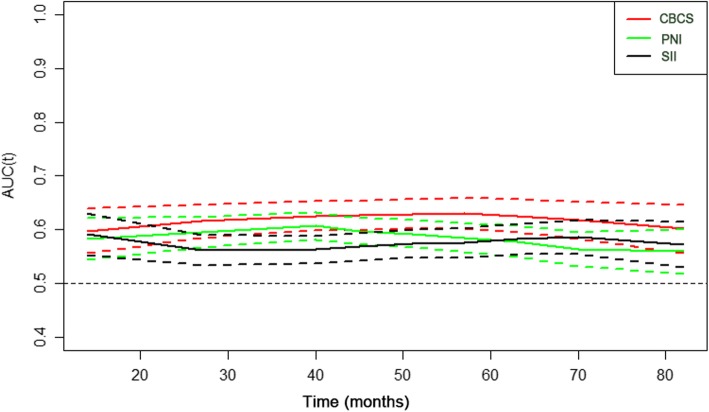
Time-dependent ROC curves of the CBCS, PNI, and SII for the prediction of overall survival. The dotted lines in Fig. 2 represent the 95% CI, and the unit of time is months. Blood samples were routinely drawn during the 7 days before surgery

We generated t-ROC curves to compare the prognostic accuracy of the CBCS and other inflammatory scoring systems (PNI, SII, mGPS and CRP/Alb). The t-ROC curve for the CBCS was consistently superior to those of the PNI and SII throughout the observation period (Fig. 2). Of the 1810 patients in our cohort with complete data, 239 also had CRP values available for analysis. The t-ROC curve for the CBCS was also superior to those for the mGPS and CRP/Alb (Additional file 2: Figure S3).

## Discussion

Currently, accumulating evidence suggests that the systemic inflammatory response plays an important role in tumor progression and metastasis [27, 28]. There has been growing interest in using CBC-based measures as biomarkers for GC [7, 8, 10]. However, there is an overlap between these indicators, and the actual prognostic accuracy of some indicators is poor. Therefore, we sought to evaluate which of these CBC-based biomarkers ultimately display the greatest potential in GC patients. The AUC is an index that can be used to compare the prognostic abilities of different factors. The higher the AUC value is, the stronger the predictive ability of the prognostic factor; this metric has been widely used in previous studies [5, 29, 30]. In this study, we compared the AUC values of similar CBC-based biomarkers, and the CBC-based biomarkers with higher AUC values were retained for further evaluation. Finally, the PLR, LMR and Hb level were retained for subsequent analyses. Multivariate analysis revealed that the preoperative LMR and Hb level were independent CBC-based predictors of OS for patients with GC undergoing curative surgical resection, which is consistent with the findings of previous studies [6, 7, 17]. Furthermore, we developed a novel CBC-based prognostic score, called the CBCS, based on the combination of the LMR and Hb level after dichotomization to more accurately and easily predict the long-term prognosis of GC patients.

This study assessed the associations between preoperative CBCS and clinicopathological factors. Our results revealed that an elevated CBCS was associated with a number of variables that were previously shown to be predictive of poor outcomes. These variables include tumor location, tumor diameter, vascular invasion, and tumor stage. In addition, multivariate analysis demonstrated that the CBCS is an independent prognostic factor for GC patients. At present, there is no consensus on how the AUC value can be used in clinical practice [9, 31, 32]. In our study, the AUC value of CBCS was 0.627, which was significantly higher than those of the LMR and Hb level according to the Delong test [33]. Our study indicated that the CBCS has better discriminatory ability than its components in terms of determining the prognosis of GC patients. As an inflammatory scoring system based on the LMR and Hb level, the biological rationale behind the prognostic value of the CBCS might involve the function of monocytes, lymphocytes and Hb. Circulating monocytes may contribute to both tumor growth and reduced immunosurveillance, which is supported by previous findings [34]. In addition, there is mounting evidence that tumor-associated macrophages (TAMs) primarily exert protumoral activity, including the promotion of metastasis, immunosuppression, and tumor angiogenesis [35]. Therefore, an increase in peripheral blood monocytes is associated with poor prognosis in patients. Lymphocytes are basic components of the adaptive and innate immune systems and form the cellular basis of immunosurveillance and immunoediting [36]. Due to tumor-infiltrating lymphocyte-induced antitumor activity and the inhibition of angiogenesis, the presence of tumor-infiltrating lymphocytes is associated with improved survival in various cancers [37]. Lymphopenia has been associated with poor prognosis in cancer patients [38, 39]. In addition, Zhang et al. demonstrated that anemia is an independent risk factor for advanced GC [40]. Anemia may have an impact on the quality of life, performance status, treatment tolerance, clinical symptoms, recovery from surgery and even outcomes [41, 42]. Therefore, the CBCS, which is based on both the LMR and the Hb level, may enable a better understanding of the effects of the tumor on both ongoing systemic inflammation and the functional state of patients. We also found that the CBCS could be used for further risk stratification in each TNM stage, suggesting that the CBCS might provide additional prognostic information to complement postoperative pathological staging.

In recent years, a number of inflammatory scoring systems, such as the SII, PNI, mGPS, and CRP/Alb, have been established to predict the prognosis of GC [9, 18, 19, 32]. In this study, we used t-ROC analyses to compare the prognostic values of CBCS and other inflammatory scoring systems, including the SII, PNI, mGPS and CRP/Alb. The advantage of this method is that it can assess the impact of individual prognostic factors and enables the analysis of survival data with censoring using ROC curves [25]. In addition, we found that the t-ROC curve for the CBCS was consistently superior to those for the SII and PNI after surgery. We also attempted to clarify the utility of the mGPS and CRP/Alb in comparison with CBCS. In our center, CRP was not a routine parameter tested in GC patients in the past. However, in recent years, it has been reported that preoperative CRP levels may affect the prognosis of GC. Thereafter, we routinely performed preoperative CRP examinations. Thus, CRP levels were only available in some patients. Our analysis demonstrated that the CBCS yielded a better t-ROC curve than did mGPS and CRP/Alb. Thus, as a novel inflammatory prognostic factor, CBCS is a superior predictor of OS compared with other inflammatory scoring systems. Furthermore, we found that compared with the traditional pathological stage, the model established by combining the CBCS and pTNM stages can predict the long-term survival of patients with GC more effectively. Thus, the CBCS can be used as a supplement to the traditional pathological stage in clinical practice to better stratify patients and provide a more accurate basis for guiding postoperative follow-up and individualized treatment.

Nevertheless, there were several limitations in our study. First, because of its retrospective nature, our study may have been subject to selection bias. For example, only some patients had preoperative CRP values. Additionally, because of the retrospective nature of the study, the time between drawing the blood and analysis of the blood sample was not noted. Second, we excluded patients with neoadjuvant therapy to ensure that all patients were in the same state before blood sampling. It is common practice in the West to give neoadjuvant treatment to patients with locally advanced disease, but this is not common practice in the East [43]. In China, most patients do not receive pre-adjuvant therapy, so there are relatively fewer patients with of neoadjuvant therapy in this study, and most patients with neoadjuvant chemotherapy have more advanced disease, usually stage T4b or metastatic disease. Therefore, the results of this study are not applicable to GC patients undergoing neoadjuvant therapy. Third, due to the lack of data from other centers, we could not validate the results externally. Last, the AUC values of CBCS are low, but after combining the CBCS with the traditional staging system, we found that the CBCS can improve the accuracy of the prognostic evaluation for patients with GC.

## Conclusions

Our study is the first to develop the CBCS and demonstrate that the preoperative CBCS, based on the LMR and the Hb level, is the most efficient marker for predicting OS in GC patients. The findings of this study can help clinicians select, as part of individualized GC treatment strategies, the most effective inflammatory markers for preoperative risk stratification and postoperative follow-up.

## Supplementary information

**Additional file 1: Table S1.** Clinicopathological characteristics. **Table S2.** Comparison of the AUCs between CBC-based parameters. **Table S3.** Definition of the complete blood count-based score (CBCS).

**Additional file 2: Figure S1.** Selection of the study population. **Figure S2.** Kaplan-Meier analysis of overall survival according to (A) the preoperative PLR, (B) the preoperative LMR, (C) the preoperative hemoglobin level, (D) the combination of the preoperative serum hemoglobin level and the LMR. **Figure S3.** Time-dependent ROC curves of the CBCS, mGPS, and CRP/Alb for the prediction of overall survival. The dotted lines in Fig. 2 represent the 95% CI, and the unit of time is months.

## Data Availability

The dataset analyzed in this study is available from the corresponding author upon reasonable request.

## Abbreviations

Alb: Albumin
ASA: American Society of Anesthesiologists
AUC: Area under the curve
BMI: Body mass index
CBCS: Complete blood count-based inflammatory score
CI: Confidence interval
CRP: C-reactive protein
GC: Gastric cancer
Hb: Hemoglobin
HR: Hazard ratio
LMR: Lymphocyte-monocyte ratio
mGPS: Modified Glasgow Prognostic Score
NLR: Neutrophil- lymphocyte ratio
OS: Overall survival
PLR: Platelet- lymphocyte ratio
SII: Systemic Immune-inflammation Index
T-ROC: Time-dependent receiver operating characteristic
