# Supplementary material for: Complete blood count-based inflammatory score (CBCS) is a novel prognostic marker for gastric cancer patients after curative resection
Source: BMC Cancer. 2020 Jan 6;20:11. doi: 10.1186/s12885-019-6466-7 (PMC6943946; doi:10.1186/s12885-019-6466-7)
Supplement: Supplementary file 1 — Additional file 1: Table S1. Clinicopathological characteristics. Table S2. Comparison of the AUCs between CBC-based parameters. Table S3. Definition of the complete blood count-based score (CBCS). [file 12885_2019_6466_MOESM1_ESM.doc]

**Additional file 1**

**Table S1.** Clinicopathological characteristics.

| Clinicopathological features | All case=1810 |
| --- | --- |
|
| Patient characteristics |  |
| Age (median,IQR) | 61(55-69) |
| Gender |  |
| Male | 1374(75.9%) |
| Female | 436(24.1%) |
| BMI (median,IQR) | 22.0(20.2-23.9) |
| ASA score |  |
| 1 | 1126(62.2%) |
| 2 | 626(34.6%) |
| 3 | 58(3.2%) |
| Disease characteristics |  |
| Tumor Location |  |
| Upper | 441(24.4%) |
| Middle | 320(17.7%) |
| Lower | 815(45.0%) |
| Mixed | 234(12.9%) |
| Tumor size (cm, median,IQR) | 4.0(3.0-6.0) |
| Histologic type |  |
| Differentiated | 362(20.0%) |
| Undifferentiated | 1448(80.0%) |
| Vascular invasion |  |
| Negative | 1406(77.7%) |
| Positive | 404(22.3%) |
| Perineural invasion |  |
| Negative | 1538(85.0%) |
| Positive | 272(15.0%) |
| pTNM stage |  |
| I | 515(28.5%) |
| II | 433(23.9%) |
| III | 862(47.6%) |
| T stage |  |
| T1 | 447(24.7%) |
| T2 | 199(11.0%) |
| T3 | 543(30.0%) |
| T4 | 621(34.3%) |
| N stage |  |
| N0 | 687(38.0%) |
| N1 | 282(15.6%) |
| N2 | 292(16.1%) |
| N3 | 549(30.3%) |
| Adjuvant chemotherapy |  |
| Yes | 1112(61.4%) |
| No | 698(38.6%) |
| CBC-based parameters |  |
| Haemoglobin (g/l), median (IQR) | 130.0(111.0-143.0) |
| Neutrophils (10^9^/l), median (IQR) | 3.6(2.8-4.6) |
| Monocytes (10^9^/l), median (IQR) | 0.4(0.3-0.5) |
| Lymphocytes (10^9^/l), median (IQR) | 1.7(1.4-2.1) |
| Platelets (10^9^/l), median (IQR) | 233.0(191.8-286.0) |
| NLR, median (IQR) | 2.1(1.5-2.9) |
| LMR, median (IQR) | 4.2(3.1-5.5) |
| PLR, median (IQR) | 135.6(102.6-183.3) |
| SII, median (IQR) | 491.0(325.0-738.3) |

**Table S2.** Comparison of the AUCs between CBC-based parameters.

| CBC-based parameters | AUC for 5-year OS |
| --- | --- |
| Neutrophils | 0.506 |
| Lymphocytes | 0.578 |
| Platelets | 0.533 |
| NLR | 0.565 |
| PLR | 0.586 |
| SII | 0.565 |
| Monocytes | 0.529 |
| LMR | 0.585 |
| Hb | 0.641 |

**Table S3.** Definition of the complete blood count-based score (CBCS).

| Scoring system | Score |
| --- | --- |
| The CBCS |  |
| LMR ≥3.4 and Hb ≥125 | 0 |
| LMR ≥3.4 and Hb <125 | 1 |
| LMR <3.4 and Hb ≥125 | 1 |
| LMR <3.4 and Hb <125 | 2 |
