# Supplementary material for: Complete blood count-based inflammatory score (CBCS) is a novel prognostic marker for gastric cancer patients after curative resection
Source: BMC Cancer. 2020 Jan 6;20:11. doi: 10.1186/s12885-019-6466-7 (PMC6943946; doi:10.1186/s12885-019-6466-7)
Supplement: Supplementary file 2 — Additional file 2: Figure S1. Selection of the study population. Figure S2. Kaplan-Meier analysis of overall survival according to (A) the preoperative PLR, (B) the preoperative LMR, (C) the preoperative hemoglobin level, (D) the combination of the preoperative serum hemoglobin level and the LMR. Figure S3. Time-dependent ROC curves of the CBCS, mGPS, and CRP/Alb for the prediction of overall survival. The dotted lines in Fig. 2 represent the 95% CI, and the unit of time is months. [file 12885_2019_6466_MOESM2_ESM.doc]

**Additional file 2**


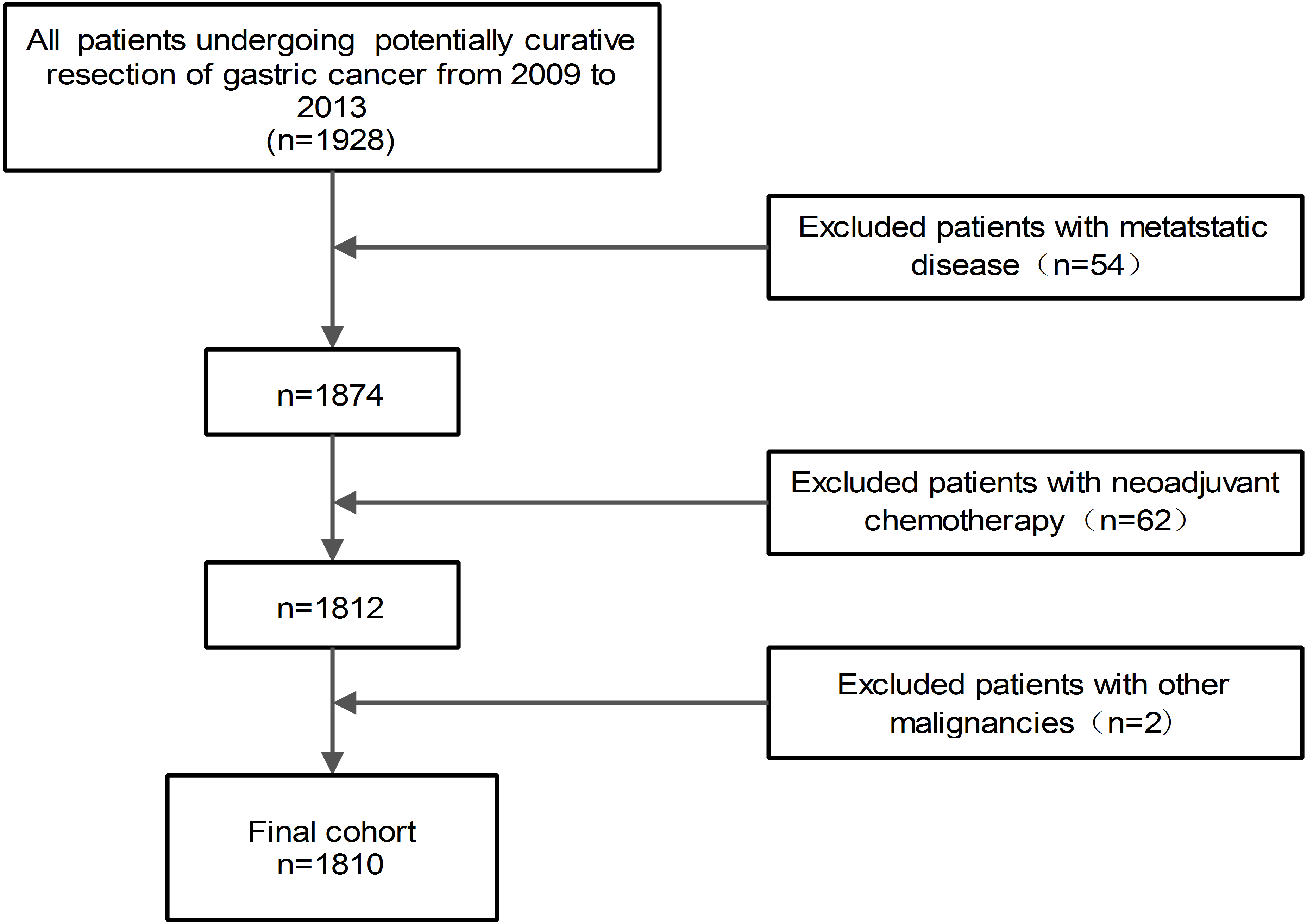


**Figure S1.** Selection of the study population.


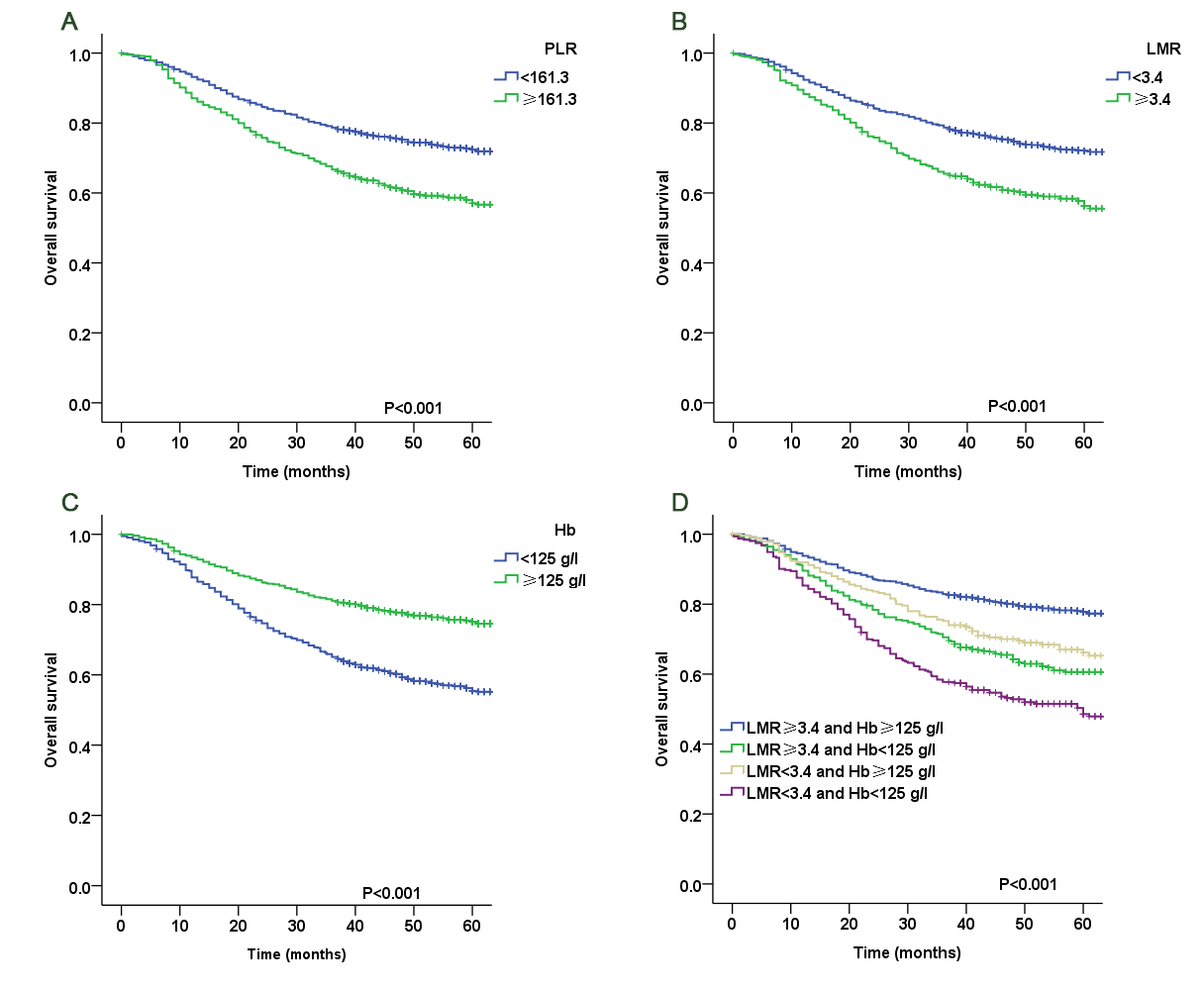


**Figure S2.** Kaplan-Meier analysis of overall survival according to (A) the preoperative PLR, (B) the preoperative LMR, (C) the preoperative hemoglobin level, (D) the combination of the preoperative serum hemoglobin level and the LMR.


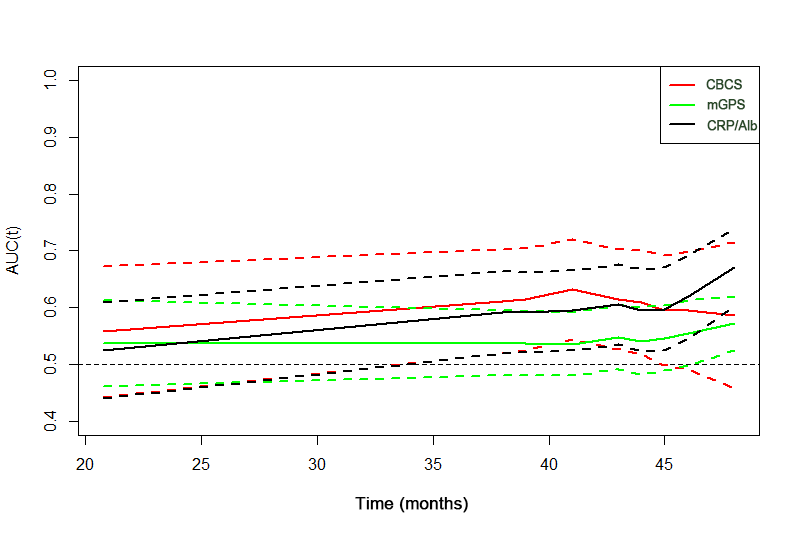


**Figure S3.** Time-dependent ROC curves of the CBCS, mGPS, and CRP/Alb for the prediction of overall survival. The dotted lines in Figure 2 represent the 95% CI, and the unit of time is months.
